# The acute effects of bilateral ovariectomy or adrenalectomy on progesterone, testosterone and estradiol serum levels depend on the surgical approach and the day of the estrous cycle when they are performed

**DOI:** 10.1186/1477-7827-6-48

**Published:** 2008-10-27

**Authors:** Angélica Flores, Alma I Gallegos, Jacqueline Velasco, Fernando D Mendoza, Cristina Montiel, Pamela M Everardo, María-Esther Cruz, Roberto Domínguez

**Affiliations:** 1 Unidad de Investigación en Biología de la Reproducción, FES Zaragoza, UNAM, Mexico

## Abstract

Bilateral ovariectomy or adrenalectomy are experimental tools used to understand the mechanisms regulating the hypothalamus-pituitary-ovarian and the hypothalamus-pituitary-adrenal axis. There is evidence that acute unilateral perforation of the dorsal peritoneum in rats results in significant changes in progesterone, testosterone and estradiol serum concentrations. Because different surgical approaches for unilateral or bilateral ovariectomy or adrenalectomy, sectioning the superior ovarian nerve or the vagus nerve are used, we compare the acute effects on hormone serum concentrations resulting from the unilateral or bilateral dorsal approach to performing bilateral ovariectomy or adrenalectomy with those obtained when an unilateral incision is performed in the ventral abdomen. In general, the progesterone, testosterone and estradiol serum concentrations were higher in animals with ventral approach than in those with dorsal surgery, the effects varying depending on the day of the estrous cycle when surgery was performed. The results suggest that the neural signals arising from different zones of the peritoneum and/or the abdominal wall play different roles in the mechanisms regulating steroid hormones concentrations.

## Background

The synthesis and release of progesterone, testosterone and estradiol is controlled by pituitary hormones [follicle stimulating hormone (FSH), luteinizing hormone (LH), adrenocorticotropin hormone (ACTH)], and non-pituitary hormones (thymulin). There is evidence that the response of hormone-secreting cells is modulated by neural information arriving to the ovaries and adrenals and presents asymmetry [1].

In previous studies we showed that in the rat, the acute unilateral perforation of the dorsal peritoneum results in significant changes in progesterone, testosterone and estradiol serum concentrations. The magnitude of these changes depended on both the side of the perforation (left or right) and the day of the estrous cycle surgery were performed. These results were interpreted as indicating a potential neural pathway between the peritoneum and the ovaries and adrenals. The information was presumed to reach the ovaries and the adrenals through their innervations, and possibly through a multisynaptic pathway [2-5].

When performing bilateral or unilateral ovariectomy or adrenalectomy, sectioning of the superior ovarian nerve or the vagus nerve, we use a ventral approach, and observed differences in hormonal responses in comparison with those observed in rats treated through the dorsal approach. The present study compares the acute effects on hormone serum concentrations of the bilateral ovariectomy or adrenalectomy performed through a dorsal approach (two incisions, left and right) or a ventral one, on each day of the estrous cycle, on progesterone, testosterone and estradiol serum concentrations.

## Methods

This study was performed with virgin adult female rats (195–225 g body weight) of the CIIZ-V strain from our own stock. Animals were kept under controlled lighting conditions (lights on from 05:00 to 19:00 h), with free access to food (Purina S.A., Mexico) and tap water. The experiments were performed following the guidelines established by The Mexican Law of Animal Protection Guidelines Treatment. The Committee of the Facultad de Estudios Superiores Zaragoza approved the experimental protocols.

Estrous cycles were monitored by daily vaginal smears. Only rats showing at least two consecutive 4-day cycles were used in the experiment. All surgeries were performed under ether anesthesia, between 13:00–13:15 h on diestrus 1 (D1), diestrus 2 (D2), proestrus (P) or estrus (E). The animals wake up immediately after surgery.

Rats were randomly allotted to one of the experimental groups described below. Animals from different experimental groups were treated simultaneously and sacrificed one hour after surgery. The number in parenthesis indicates the number of animals in each group.

### Experimental groups

#### Control group

Non-treated cyclic rats sacrificed at 14:00 h on D1 (29), D2 (30), P (25) or E (18).

#### Anesthetized group

Rats were anesthetized with ether at 13:00 h on D1 (20), D2 (20), P (19) or E (20).

#### Sham operation – dorsal approach

A bilateral incision (left and right side) was performed 2 cm below the last rib; affecting skin, muscle, and peritoneum [D1 (11), D2 (10), P (11) or E (10)]. No organs were extirpated. The wound was subsequently sealed.

#### Sham operation – ventral approach

An incision was performed 2 cm below the sternum; affecting skin and muscle and peritoneum [D1 (9), D2 (10), P (10) or E (10)]. No organs were extirpated or handled. The wound was subsequently sealed.

#### Bilateral ovariectomy or adrenalectomy – dorsal approach

A bilateral (left and right) incision, including skin, muscle, and peritoneum were performed 2 cm below the last rib, and the left and right ovaries or left and right adrenals were extirpated [ovariectomy D1 (10), D2 (10), P (9) or E (10)]; [adrenalectomy D1 (10), D2 (12), P (10) or E (10)]. The wound was subsequently sealed.

#### Bilateral ovariectomy or adrenalectomy – ventral approach

An incision was performed 2 cm below the sternum and extended to the right, left or both sides of the abdominal wall; affecting skin, muscle, and peritoneum. The ovaries or adrenals were extirpated [ovariectomy D1 (10), D2 (10), P (9) or E (10)] [adrenalectomy D1 (10), D2 (10), P (10) or E (9)]. The wound was subsequently sealed.

### Autopsy procedures

Rats were sacrificed by decapitation one hour after treatment. The blood of the trunk was collected, allowed to clot at room temperature for 30 minutes, and centrifuged at 3,000 rpm during 15 minutes. Serum was stored at -20°C, until P_4_, T and E_2 _concentrations were measured.

### Hormone assay

Concentrations of P_4_, T and E_2 _in serum were measured by Radio-Immuno-Assay (RIA) using kits purchased from Diagnostic Products (Los Angeles, CA). Results are expressed in ng/ml (progesterone) and pg/ml (testosterone and estradiol). The Intra- and inter-assay variation coefficients were 5.3% and 9.87% for progesterone, 5.6% and 8.7% for testosterone and 6.9% and 10.8% for estradiol.

### Statistics

Differences in serum hormone concentrations between the two groups were analyzed using the Student's t-test. Data on hormonal concentrations in serum were analyzed using the multivariate analysis of variance (MANOVA) followed by Tukey's test. A probability value of less than 5% was considered significant.

## Results

### Differences in hormone levels in rats with dorsal or ventral peritoneum perforation

Progesterone levels in rats treated by a dorsal approach on D2 were higher than in control group. Ventral peritoneum perforation performed on D1, D2, P or E day resulted in significantly higher progesterone levels than control. In those animals treated on D1, P or E it was also significantly higher than animals treated by a dorsal approach [D1 63.5 ± 11.1 vs. 26.5 ± 1.1; P 28.7 ± 2.4 vs. 16.3 ± 1.6; E 26.3 ± 1.1 vs. 14.7 ± 1.9, p < 0.05].

For testosterone, dorsal treated animals at the day of P showed higher testosterone serum levels than in control rats. Those rats submitted to ventral surgery on D2, P and E showed higher levels than in control rats. In comparison with the dorsal treated animals on D2 or E, testosterone serum levels were significantly higher in ventral treated rats [D2 103.4 ± 14 vs. 26.0 ± 6.5; E 76.5 ± 20.8 vs. 2, p < 0.05].

Estradiol levels in rats treated on P by a dorsal surgery were lower than in control group. Ventral approach did not modify estradiol levels in comparison with control group. In rats treated in D1 or D2, the ventral approach resulted in lower estradiol levels than those treated by the back, while they were higher when the animals were treated on P [D1 46.6 ± 5.6 vs. 76.3 ± 6.5; D2 38.6 ± 2.8 vs. 63.3 ± 7.1; P 149.7 ± 11.3 vs. 93.7 ± 16.8, p < 0.05] 1.

**Table 1:** Progesterone, testosterone and estradiol levels in control rats and animals treated with anesthesia, ventral or dorsal sham surgery performed at 13:00 h on each day of the estrous cycle and sacrificed 1 h after treatment

Group	Diestrus 1	Diestrus 2	Proestrus	Estrus
PROGESTERONE				
Control	22.9 ± 1.9	8.0 ± 1.1	11.1 ± 1.9	17.3 ± 2.4
Anesthesia	41.6 ± 6.0*	14.9 ± 3.4	16.9 ± 2.3	11.3 ± 1.5
Sham ventral	63.5 ± 11.1*◆	33.8 ± 4.4◆*	28.7 ± 2.4◆*	26.3 ± 1.1*◆
Sham dorsal	26.5 ± 1.1^#^	21.7 ± 2.1*	16.3 ± 1.6^#^	10.9 ± 2.1
TESTOSTERONE				
Control	8.5 ± 2.3	54.5 ± 8.4	99.0 ± 14.1	<2
Anesthesia	7.0 ± 2.6	86.8 ± 13.5	132.0 ± 22.4	<2
Sham ventral	19.1 ± 8.6*◆	103.4 ± 14.0*	285.8 ± 28.0*◆	76.5 ± 20.8*◆
Sham dorsal	8.6 ± 2.9^#^	26 ± 6.5^#^◆	206 ± 16.1*	<2^#^
ESTRADIOL				
Control	57.3 ± 6.0	49.1 ± 5.0	144.0 ± 11.8	22.5 ± 2.9
Anesthesia	45.7 ± 6.2	54.5 ± 7.7	142.2 ± 17.2	20.4 ± 2.1
Sham ventral	46.6 ± 5.6	38.6 ± 2.8	149.7 ± 11.3	30.2 ± 2.6
Sham dorsal	76.3 ± 6.5	63.3 ± 7.1	93.7 ± 16.8	28.8 ± 4.0

*p < 0.05 vs. Control; ◆ vs. Anesthesia; ^# ^vs. Sham ventral. MANOVA followed by Tukey's test

Because the perforation of the peritoneum resulted in significant differences with control animals and depended on the side and day of the cycle when it was performed, the effects of bilateral ovariectomy or adrenalectomy were compared with proper sham operated animals.

### Hormone levels in bilateral ovariectomized and adrenalectomized rats

#### Progesterone (Figure 1) – ventral approach

**Figure 1:**
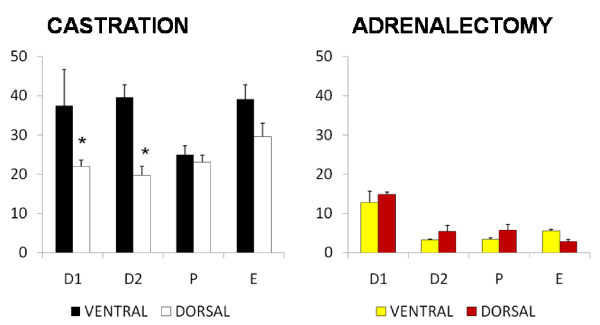
**Effect of castration or adrenalectomy by ventral or dorsal approach on progesterone serum levels (ng/ml)**. Means ± SEM of progesterone serum concentration in sham operated rats, bilateral ovariectomized (CAS) and bilateral adrenalectomized animals (ADX) by ventral or dorsal approach *p < 0.05 vs. control

The progesterone levels in rats with bilateral ovariectomy performed on D1 were lower than in sham operated animals. In those animals treated on D2, P or E progesterone levels were similar to sham operated animals. Bilateral adrenalectomy resulted in significantly lower progesterone serum levels than in sham and castrated animals.

#### Dorsal approach

Bilateral ovariectomy performed on P or E resulted in higher progesterone levels than in sham operated animals. Bilateral adrenalectomy resulted in significantly lower progesterone serum levels than in sham and castrated animals.

#### Testosterone (Fig. 2) – Ventral approach

**Figure 2:**
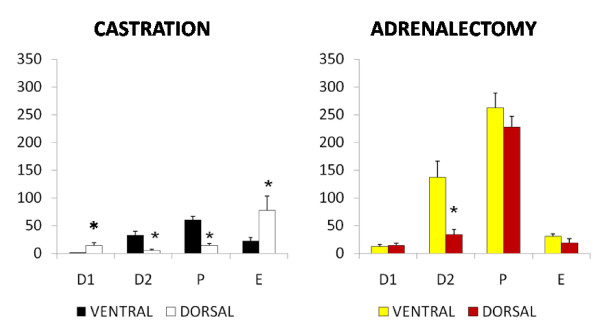
**Effect of castration or adrenalectomy by ventral or dorsal approach on testosterone serum levels (pg/ml)**. Means ± SEM of testosterone serum concentration in sham operated rats, bilateral ovariectomized (CAS) and bilateral adrenalectomized animals (ADX) by ventral or dorsal approach *p < 0.05 vs. control; ^#^p < 0.05 vs. CAS.

Bilateral ovariectomy performed on each day of the estrous cycle resulted in lower testosterone levels than in sham operated animals. Bilateral adrenalectomy on E resulted in lower testosterone serum levels than in sham operated animals, while when it was performed on D1, D2 or P testosterone concentrations were similar to sham operated animals.

#### Dorsal approach

Castration performed on D2 or P resulted in lower testosterone levels than in sham operated animals, while the concentration of the hormone was higher when it was performed on E. Bilateral adrenalectomy performed on E resulted in higher testosterone levels than in sham operated group, while significantly differences in rats treated on D1, D2 or P, were not observed.

#### Estradiol (Figure 3) – ventral approach

**Figure 3:**
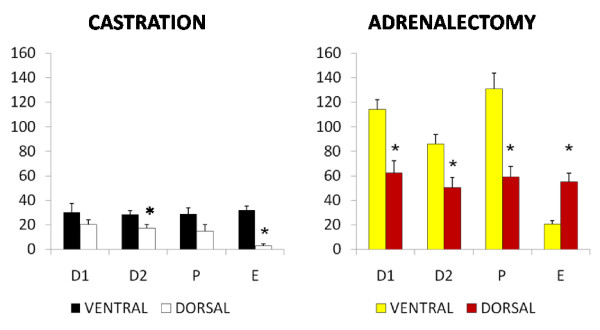
**Effect of castration or adrenalectomy by ventral or dorsal approach on estradiol serum levels (pg/ml)**. Means ± SEM of estradiol serum concentration in sham operated rats, bilateral ovariectomized (CAS) and bilateral adrenalectomized animals (ADX) by ventral or dorsal approach. *p < 0.05 vs. control; ^#^p < 0.05 vs. CAS.

Bilateral ovariectomy on P resulted in lower estradiol serum levels than in sham operated group. Bilateral adrenalectomy performed on D1 or D2 resulted in higher estradiol serum levels than in sham operated animals.

#### Dorsal approach

Bilateral ovariectomy resulted in lower estradiol serum levels than in sham operated group. Bilateral adrenalectomy performed on P resulted in lower estradiol serum levels than in sham operated animals, while an inverse result occurred when the animals were treated on E.

## Discussion

The results presented herein indicate that the adrenals are the main source of progesterone during the estrous cycle, since bilateral ovariectomy did not modify the increased progesterone levels resulted from the sham operation, while bilateral adrenalectomy resulted in a significant decrease in progesterone concentration. They also suggest that the ovaries are the main source of testosterone and estradiol during the cycle, and the lost of adrenal signals as result of bilateral adrenalectomy affects testosterone and estradiol levels depending on the surgical approach and the day of the cycle when surgery was performed. Such results suggest that in the mechanism regulating ovarian steroid hormone secretion the neural information arising from the dorsal and ventral peritoneum play different roles. In addition, the adrenal hormones and/or neural signals arising from the gland play a different role in the regulation of estradiol secretion depending on both the surgical approach and the day of the estrous cycle studied.

The results indicate that the participation of the proposed neural pathways in the regulation of ovarian secretion is not similar for the three hormones studied. Because the hormone level depends on both the secretion and excretion rates, we cannot exclude the possibility that the neural information arising from different zones of the peritoneum modulates in different ways both process for each hormone.

Another possibility is that the peripheral neural pathways modulating the hormone secretion/excretion process also arise from the skin and muscles affected during the surgery. In this regard, Stener-Victorin et al. [6,7] using electro-acupuncture (EA) for the treatment of polycystic ovarian syndrome (PCOS) have shown that repeated EA treatments in rats with steroid-induced PCOs, modulates nerve growth factor concentration in the ovaries and corticotropin releasing factor in the median eminence. Endothelin-1 concentration in the ovaries was significantly lower in the PCOS group receiving EA compared with the control group, while in the hypothalamus its concentration was significantly higher in the PCOS group receiving EA than in the healthy control group. They conclude that EA modulates the neuroendocrinological state of the ovaries, by modulating the sympathetic nerve activity in the ovaries, which may be a factor in the maintenance of steroid-induced PCO.

There is strong evidence that stress increases the activity of the hypothalamic-pituitary-adrenal (HPA) axis and decreases reproductive functions, through the increase in CRF that in turn stimulates ACTH and β-endorphin secretion, while ACTH stimulates the adrenal corticosteroids secretion. Stener-Victorin et al. [8] suggest that via this route, EA may exert an effect on both the HPA axis and the hypothalamic-pituitary-gonadal axis. In another study, Stener-Victorin et al. [9] have shown that the ovarian blood flow response to both abdominal and hind limb electro-acupuncture stimulation is mediated as a reflex response via the ovarian sympathetic nerves, and the response is controlled via supraspinal pathways. The ovarian blood flow responses to segmental abdominal EA stimulation are also frequency dependent and are amplified during the estrous phase.

In a previous study [3], the superimposition of two stressors, ether anesthesia and perforation of the dorsal peritoneum, did not result in higher increase of progesterone levels secretion than those induced only by the anesthesia. This result suggests that the response capacity to stress by the hypothalamus-pituitary-adrenal axis, manifested by increasing progesterone secretion, reaches its peak with the effects of ether anesthesia. According to the present data, we observed that ventral peritoneum perforation resulted in higher progesterone, testosterone and estradiol levels than in ether anesthetized animals (data not shown), again depending on the day of the estrous cycle studied. In rats treated on D1, D2 and E, the perforation of the ventral peritoneum resulted in higher progesterone levels than those in animals treated through the back. These results could indicate the existence of different neural communications between the ventral and dorsal abdominal wall and/or peritoneum with control systems in the central nervous system.

Perhaps the neural communication resulting from the ventral perforation of the peritoneum and/or abdominal wall resulted in an increased release of LH and/or ACTH, which stimulates the synthesis and release of progesterone, reflected in the higher concentration of the hormone detected in serum. This mechanism was proposed in earlier studies to explain the increases in progesterone levels observed in animals with bilateral peritoneum perforation performed on D2 [3].

Ether anesthesia treatment to rats in proestrus day resulted in significant increases in testosterone serum levels, while the same treatment on D1 or D2 had no significant effects. On P day, perforation of the dorsal peritoneum on the left side resulted in a significant increase in testosterone serum levels, while perforation of the right side caused no significant changes in hormone levels [3]. Such results were interpreted as a support of a previous proposal, that neural mechanisms regulating testosterone secretion by the ovaries, or the synthesis of testosterone by non-gonadal organs is asymmetric [4]. The idea of different neural pathways modulating hormone secretion is supported by the differences in testosterone levels between rats with perforation of the ventral peritoneum and animals with dorsal perforation of the peritoneum. Independently of the day of the estrous cycle when the animals treated, testosterone concentrations in rats with ventral peritoneum perforation were significantly higher than those in animals with dorsal peritoneum perforation. In women, 25% of testosterone production is secreted by the ovaries, 25% by the adrenals, and 50% arise from the peripheral metabolism of pre-hormones [10]. According to Stahl et al. [10], the gonads are the main sources of testosterone in female rats, while the contribution of the adrenals to plasma testosterone levels is only moderate and much smaller than in human beings. Present results support such idea. We can presume, therefore, that neural signals arising from the ventral abdominal wall and/or peritoneum arrive to the ovaries and participate in modulating testosterone secretion.

## Conclusion

Taken together, present and previous results support the idea that neural signals arising from different zones of the peritoneum, abdominal wall and other organs, participate in the mechanisms regulating steroid hormones levels.

## Competing interests

The authors declare that they have no competing interests.

## Authors' contributions

AF and RD conceived and designed the study. AIG, JV, FDM, CM, PME performed the surgeries and hormone measurements. MEC participated in the analysis and discussion of the results. RD prepared the initial draft of the manuscript. All co-authors provided inputs during final manuscript preparation. All authors read and approved the final manuscript.
